# “*Candidatus* Chlorobium masyuteum,” a Novel Photoferrotrophic Green Sulfur Bacterium Enriched From a Ferruginous Meromictic Lake

**DOI:** 10.3389/fmicb.2021.695260

**Published:** 2021-07-09

**Authors:** Nicholas Lambrecht, Zackry Stevenson, Cody S. Sheik, Matthew A. Pronschinske, Hui Tong, Elizabeth D. Swanner

**Affiliations:** ^1^Department of Geological and Atmospheric Sciences, Iowa State University, Ames, IA, United States; ^2^Department of Biology, University of Minnesota Duluth, Duluth, MN, United States; ^3^Large Lakes Observatory, University of Minnesota Duluth, Duluth, MN, United States; ^4^Guangdong Key Laboratory of Integrated Agro-environmental Pollution Control and Management, National-Regional Joint Engineering Research Center for Soil Pollution Control and Remediation in South China, Guangdong Institute of Eco-environmental Science and Technology, Guangdong Academy of Sciences, Guangzhou, China

**Keywords:** photoferrotrophy, Brownie Lake, meromictic, green sulfur bacterium, phototrophic Fe(II) oxidation, early Earth biogeochemistry, iron cycling, geomicrobiology

## Abstract

Anoxygenic phototrophic bacteria can be important primary producers in some meromictic lakes. Green sulfur bacteria (GSB) have been detected in ferruginous lakes, with some evidence that they are photosynthesizing using Fe(II) as an electron donor (i.e., photoferrotrophy). However, some photoferrotrophic GSB can also utilize reduced sulfur compounds, complicating the interpretation of Fe-dependent photosynthetic primary productivity. An enrichment (BLA1) from meromictic ferruginous Brownie Lake, Minnesota, United States, contains an Fe(II)-oxidizing GSB and a metabolically flexible putative Fe(III)-reducing anaerobe. “*Candidatus* Chlorobium masyuteum” grows photoautotrophically with Fe(II) and possesses the putative Fe(II) oxidase-encoding *cyc2* gene also known from oxygen-dependent Fe(II)-oxidizing bacteria. It lacks genes for oxidation of reduced sulfur compounds. Its genome encodes for hydrogenases and a reverse TCA cycle that may allow it to utilize H_2_ and acetate as electron donors, an inference supported by the abundance of this organism when the enrichment was supplied by these substrates and light. The anaerobe “*Candidatus* Pseudopelobacter ferreus” is in low abundance (∼1%) in BLA1 and is a putative Fe(III)-reducing bacterium from the *Geobacterales* ord. nov. While “*Ca.* C. masyuteum” is closely related to the photoferrotrophs *C. ferroooxidans* strain KoFox and *C. phaeoferrooxidans* strain KB01, it is unique at the genomic level. The main light-harvesting molecule was identified as bacteriochlorophyll *c* with accessory carotenoids of the chlorobactene series. BLA1 optimally oxidizes Fe(II) at a pH of 6.8, and the rate of Fe(II) oxidation was 0.63 ± 0.069 mmol day^–1^, comparable to other photoferrotrophic GSB cultures or enrichments. Investigation of BLA1 expands the genetic basis for phototrophic Fe(II) oxidation by GSB and highlights the role these organisms may play in Fe(II) oxidation and carbon cycling in ferruginous lakes.

## Introduction

Iron is a major redox-active element on Earth (Raiswell and Canfield, 2012). The biogeochemical cycling between the two main redox states, Fe(II) and Fe(III), is accomplished by both aerobic and anaerobic microbes, as well as abiotic chemical reactions (Melton et al., 2014). Active redox cycling mediated by microbes at the interface of oxic and anoxic settings couples the Fe biogeochemical cycles at Earth’s surface to that of several other major elemental cycles (e.g., C, O, S, N; Kappler et al., 2021), underscoring the necessity to elucidate microbiological pathways that transform Fe and the controls on their activity in the environment.

Investigation of modern Fe cycling organisms may also help to constrain microbial processes in Precambrian (i.e., >540 million years ago; Ma) oceans, which were characterized by widespread and persistent ferruginous (anoxic and Fe-rich) conditions (Poulton and Canfield, 2011). Prior to the development of oxygenated surface waters after the Great Oxidation Event (GOE) at ∼2.4 billion years ago (Ga), anoxygenic photosynthetic bacteria (APB) that could utilize Fe(II) in the photic zone may have been the major marine primary producers fueling the biosphere in the Archean (4.0–2.5 Ga), sustaining up to 10% of modern-day primary productivity prior to the evolution of oxygenic photosynthesis by Cyanobacteria (Canfield et al., 2006; Jones et al., 2015). These organisms, collectively known as photoferrotrophs, are bacteria that use light energy, Fe(II) as an electron donor, and inorganic carbon to perform anoxygenic photosynthesis (Ehrenreich and Widdel, 1994; Kappler et al., 2005):

(1) $$\text{light}\,\left(h\,v\right)+4\,F\,e^{2+}+C\,O_{2}+11\,{\text{H}}_{2}\,\text{O}\to 4\,F\,e\,{\left(\text{OH}\right)}_{3}+\left({\text{CH}}_{2}\,\text{O}\right)+8\,H^{+}$$

Photoferrotrophs have been implicated as major contributors to primary productivity in ferruginous Kabuno Bay of Lake Kivu (Llirós et al., 2015; Morana et al., 2016). They fix carbon in ferruginous Lake Svetloe (Savvichev et al., 2017). In ferruginous Lake La Cruz, photoferrotrophic activity was detected despite these organisms being only a small fraction of the APB community (Walter et al., 2014). However, the presence of sunlight and ferruginous conditions are not strong indicators that photoferrotrophy is occurring or is biogeochemically significant; increasing genomic evidence suggests photoferrotrophic APB are potentially widespread and active in many lakes (Tsuji et al., 2020; Garcia et al., 2021).

Photoferrotrophs are phylogenetically diverse and include the classes Alphaproteobacteria (purple non-sulfur bacteria, PNSB), Gammaproteobacteria (purple sulfur bacteria, PSB), and the family *Chlorobiaceae* within the class Chlorobia (green sulfur bacteria, GSB). Isolates or defined co-cultures of photoferrotrophs include the freshwater PNSB *Rhodobacter ferrooxidans* strain SW2 (Ehrenreich and Widdel, 1994) and *Rhodopseudomonas palustris* strain TIE-1 (Jiao et al., 2005); the PSB include the freshwater *Thiodictyon* sp. (Ehrenreich and Widdel, 1994; Croal et al., 2004) and the marine *Rhodovulum robiginosom* and *Rhodovulum iodosum* (Straub et al., 1999); and the GSB include the marine *Chlorobium* sp. strain N1 enrichment (Laufer et al., 2017; Bryce et al., 2019), the freshwater *Chlorobium ferrooxidans* strain KoFox, which grows in coculture with *Geospirillum* sp. strain KoFum (Heising et al., 1999), the freshwater coculture of a strain closely related to *Chlorobium ferrooxidans* that grows in coculture with a strain closely related to *Rhodopseudomonas palustris* (Schmidt et al., 2021), and the isolate *Chlorobium phaeoferrooxidans* strain KB01 (Crowe et al., 2017). *C. phaeoferrooxidans* strain KB01 is the first photoferrotroph to be isolated from a ferruginous water column (Llirós et al., 2015).

In addition to Fe(II) oxidation, some APB can exploit sulfide as a photosynthetic electron donor (Straub et al., 1999; Laufer et al., 2017). APB utilize bacteriochlorophyll (Bchl) molecules and accessory pigments such as carotenoids to harvest light energy. Previous studies have documented the presence of Bchl *e* and increased methylation of hopanoids under ferruginous conditions and attributed their presence to photoferrotrophic activity (Eickhoff et al., 2013; Crowe et al., 2014). The detection of degradation products of such biomolecules (e.g., biomarkers) in ancient rocks has been used as evidence for euxinic conditions, i.e., free sulfide present, in the Phanerozoic (e.g., Summons and Powell, 1986; Grice et al., 2005; Mallorquí et al., 2005; Hays et al., 2007) and Paleoproterozoic oceans (e.g., Brocks et al., 2005; Brocks and Schaeffer, 2008). However, specific pigments do not always indicate the electron donor being used [i.e., Fe(II) or sulfide] and, by implication, ferruginous or euxinic environmental conditions. Rather, the presence of these biomarkers establishes paleoenvironments in which light reached anoxic portions of ancient water columns, inferring the presence of obligate anoxygenic phototrophs. To fully understand and interpret the biomarker record, identification of potential biomarkers produced by diverse modern photoferrotrophs, especially those active under ferruginous conditions, is required.

Here, we describe the enrichment and characterization of a novel pelagic Fe(II)-oxidizing photoferrotroph from a meromictic and ferruginous lake that is closely related to other phototrophic GSB, but distinct in genomic and physiological characteristics. Similar to other photoferrotrophic GSB (Heising et al., 1999; Bryce et al., 2019; Schmidt et al., 2021), this organism could not be isolated, but is the most abundant organism in the enrichment. The characterization of novel photoferrotrophs informs the framework for understanding how APB, specifically GSB, influence carbon and iron cycling within ferruginous systems. The organism described here is only the second photoferrotroph to be brought into enrichment from a ferruginous water column, which highlights the difficulties, but also value, in bringing these organisms into culture.

## Materials and Methods

### Enrichment and Cultivation

Freshwater from Brownie Lake in Minneapolis, Minnesota, United States, was taken from the chemocline (5.5 m) in May 2016. The field site and geochemical conditions have been described previously (Lambrecht et al., 2018). Site water (1 ml) was added to Hungate tubes containing 9 ml of freshwater (FW) medium amended with anoxic ferrous chloride (FeCl_2_).

The FW medium for enrichment and cultivation had a salinity of 1.6. Per liter, the medium contained the following salts: 0.14 g KH_2_PO_4_, 0.3 g NH_4_Cl, 0.5 g MgSO_4_ × 7H_2_O, and 0.1 g CaCl_2_ × 2H_2_O. The medium was degassed under N_2_/CO_2_ (90:10 v/v) and buffered with 22 mM NaHCO_3_ (1.85 g l^–1^). Lastly, the following supplements were added in 1-ml volumes per liter: vitamin solution (1 mg biotin, 10 mg nicotinate, 5 mg aminobenzoic acid, 2.5 mg Ca-D(+) pantothenate, 25 mg pyridoxamine dihydrochloride, 5 mg thiamine dihydrochloride, and 100 mg vitamin B_12_ in 100 ml Millipore water), selenium-tungstate solution (0.4 g NaOH, 6 mg Na_2_SeO_3_ × 5H_2_O, and 8 mg Na_2_WO_4_ × 2H_2_O in 1 l Millipore water), and a trace element solution (10 ml of 25% HCl, 2.86 g H_3_BO_3_, 0.5 g MnCl_2_ × 4H_2_O, 180 mg ZnCl_2_, 36 mg Na_2_MoO_4_ × 2H_2_O, 2 mg CuCl_2_ × 2H_2_O, 24 mg NiCl_2_ × 6H_2_O, 190 mg CoCl_2_ × 6H_2_O, and 1.5 g FeCl_2_ × 4H_2_O in 1 l Millipore water). The pH was adjusted using sterile anoxic 1 M HCl or 0.5 M Na_2_CO_3_ after autoclaving. Anoxic FeCl_2_, prepared according to Hegler et al. (2008), was subsequently added (∼3 mM final conc.), and precipitation with phosphate and carbonate was allowed to occur at 4°C for 48 h. Following precipitation, the media was filtered (0.2 polyethersulfone filter) in an anoxic chamber (100% N_2_), dispensed in serum bottles, and the headspace promptly exchanged for N_2_/CO_2_ (90:10 v/v).

Enrichment was done at 20°C with illumination from two fluorescent bulbs, one warm (2,700 K) and one cool (5,000 K), to provide the full spectrum of photosynthetically active radiation (PAR). Delivery of the full PAR spectrum from the two fluorescent bulbs was verified with a MSC15 spectral light meter (Gigahertz-Optik). A long-pass light filter (Edmund Optics) was initially used to allow only wavelengths longer than 700 nm to the tubes to exclude growth by oxygenic photosynthesis. After several months and four transfers, a series of two serial dilutions to extinctions were performed in an effort to isolate a single organism from the enrichment. A serial 10-fold dilution series prepared up to a dilution of 10^–9^. The last dilution showing evidence of Fe(II) oxidation was transferred. Following dilution to extinction, and for all experiments subsequently described, the enrichment was incubated with the full PAR spectrum. For standard cultivation, a 4% inoculum was used.

### DNA Sequencing and Bioinformatic Processing

To identify which organisms were present in the enrichment before dilution to extinction, DNA was extracted using the DNeasy PowerSoil Kit (Qiagen) according to the instructions of the manufacturer. Near full-length 16S rRNA gene sequences were obtained using the universal bacterial primers 27F (5′-AGAGTTTGATCCTGGCTCAG-3′) and 1492R (5′-TA CGGYTACCTTGTTACGACTT-3′) (Lane, 1991). The PCR products were cloned into a plasmid vector with an ampicillin-resistant marker using the TOPO TA Cloning Kit (Thermo Fisher). Vectors were transformed into One Shot Top10 competent cells ThermoFisher (Waltham, MA, United States) that were afterward plated onto the LB medium containing ampicillin. Colonies were picked and tested for their correct size using the M13F (5′-CAGGAAACAGCTATGAC-3′) and M13R (5′-GTAAAACGACGGCCAG-3′) primer pair. The PCR product with the correct insert was purified with the PureLink PCR purification kit (Invitrogen). Sanger sequencing was performed on an Applied Biosystems 3730xl DNA Analyzer at the ISU DNA Facility. All clones sequenced were aligned with ClustalW (Thompson et al., 2003).

For genome sequencing after dilution to extinction, DNA was extracted using the DNeasy PowerSoil Kit (Qiagen, Germantown, MD, United States) and sent to the University of Minnesota Genomics Core for sequencing. Sequencing libraries were created using the Nextera-XT kit (Illumina) and sequenced on the MiSeq platform with 2 × 300 bp paired-end sequencing. Prior to assembly, raw reads were trimmed of adapters and screened for quality with FastP (Chen et al., 2018). The cleaned reads were assembled with SPAdes v. 3.14.0 (Nurk et al., 2013). Genome quality was assessed using Quast (Gurevich et al., 2013). Searches of 16S rRNA genes in the assembly with Barrnap (Seemann, 2018) revealed that the culture was not axenic and required genome binning. The assembly was binned with MetaBat1, Metabat2, and Maxbin2 (Kang et al., 2015; Wu et al., 2016; Kang et al., 2019). DASTool was used to select the highest-quality bins from the assemblies (Sieber et al., 2018). Genomes were assessed for completeness with CheckM (Parks et al., 2015) and taxonomy using GTDB-Tk (Chaumeil et al., 2019). The recovered genomes were genes called and annotated with MetaErg (Dong and Strous, 2019) and DRAM (Shaffer et al., 2020). FeGenie, a bioinformatics tool used to identify genes associated with Fe cycling, was used to screen the recovered genomes in the BLA1 enrichment for genes implicated in Fe cycling, specifically Fe(II) oxidation and Fe(III) reduction (Garber et al., 2020). The distance allowed between genes to be identified as a cluster was set to 5. A phylogenomic tree of the dominant recovered genomes with other closely related genomes was generated using GToTree (Lee, 2019). Genomes were downloaded from GTDB, and single-copy genes were identified with Hmmer^1^. Sixteen single-copy genes were used to assess the phylogenomic association (see Hug et al., 2016), as they have been shown to be robust for phylogenetic relationships. Prior to concatenation, the single-copy genes were individually aligned with Muscle (Edgar, 2004) and trimmed with trimAL (Capella-Gutiérrez et al., 2009). A maximum likelihood phylogenetic tree was created with IQ-TREE using default parameters (Nguyen et al., 2015). Genome relatedness of the *Chlorobia* genomes was assessed using Average Nucleotide Identity (ANI; Jain et al., 2018) and Digital DNA–DNA hybridization (DDH) with Genome-to-Genome Distance Tool (GGDC) (Meier-Kolthoff et al., 2013). The two genomes from this study were deposited to the National Center for Biotechnology Information (NCBI, Bioproject PRJNA611822) and the sequences to the sequence read archive (SRA; SRR11292469).

### Characterization of Growth Substrates

#### Screening of Electron Donors

The enrichment was tested on the following electron donors for anoxygenic phototrophic growth: acetate, hydrogen (H_2_) gas, hydrogen sulfide, thiosulfate, and sulfite. Acetate was added at a final concentration of 5 mM. Hydrogen gas was supplied to the culture by flushing the headspace every second day with H_2_/CO_2_ gas (90:10 v/v). The final concentrations of inorganic electron donors were as follows: sodium sulfide (2 mM), sodium thiosulfate (2 mM), and sodium sulfite (2 mM). Spectrophotometry at 600 nm was used to assess growth of the enrichment on alternative substrates.

#### Growth Experiments With Fe(II)

Fe(II) oxidation was tracked in two different types of experiments on the enrichment. The first was using triplicate bottles at different pH to determine the pH optimum. For this, three 50-ml serum bottles were filled with 25 ml of Fe(II) medium at each of the five initial pH conditions(6.7, 6.8, 6.9, 7.0, and 7.1) set with the bicarbonate-N_2_/CO_2_ buffer system and capped with rubber butyl stoppers. PAR intensity was measured with a LI-190R quantum sensor coupled to a LI-250A light meter (LiCOR). Cultures were kept in a 20°C incubator at an intensity of ∼4 μM photons m^–2^ s^–1^. Fe(II) was assessed on unfiltered samples acidified directly into 1 N HCl and measured promptly with a ferrozine assay following a protocol adapted from Stookey (1970) and Viollier et al. (2000) using an Epoch 2 Microplate Reader (Biotek). The starting concentration of Fe(II) was ∼2.5 mM.

To determine cell-specific Fe(II) oxidation rates, triplicate 100-ml bottles were filled with 50 ml Fe(II) medium adjusted to pH 6.8. Controls included dark conditions with cells and light without cells (control for photo-oxidation). Fe(II) concentrations were tracked as above. For growth quantification, samples were extracted and immediately fixed with paraformaldehyde (final conc. 3.8%) and stored at 4°C. Fe-oxide digestion and filtration onto black filters were conducted following Wu et al. (2014). Cells were stained with Sytox (1:50 dilution). Fluorescent images were collected using a Leica DFC7000T microscope. Twenty fields of view or 1,000 cells were enumerated for each replicate at each time point. Cell counting was aided by the 3D Objects Counter from ImageJ (Abràmoff et al., 2004). The maximum rate of Fe(II) oxidation was calculated for each replicate by linear regression of the steepest part of their respective Fe(II) vs. time plots (minimum three points).

### Photosynthetic Pigment Identification

#### Pigment Extraction

Pigment extraction was performed following an in-house protocol created by the Metabolomics Lab at ISU based on published protocols (Costas et al., 2012; Bóna-Lovász et al., 2013). The enrichment was grown with ferrous iron, in stationary phase, and was centrifuged at 1,000 rpm for 15 min followed by removal of the supernatant. An acetone/methanol solution (7:2 v/v) was added to the spun-down cells and sonicated in a water bath for 10 min, then vortexed for 10 min at maximum speed. After centrifugation at 1,000 rpm for 10 min, the supernatant containing the pigments was transferred to a clean tube and dried under a constant stream of N_2_ gas. Once dried, concentrated pigments were resuspended in ∼0.4 ml of the acetone/methanol solution.

#### UHPLC and MS(n) Analysis

Concentrated Bchl compounds were detected and quantified using the Agilent 6540 UHD Q-TOF LC/MS system. Chromatographic separation was performed using an Agilent 1290 Infinity series UHPLC, equipped with a diode array detector. A Zorbex Eclipse Plus C18 RRHD column (2.1 × 100 mm, 1.8 μm) was used to separate the isolated sample. Temperature during separation was held at 45°C. Analysis was performed using a 10-μl injection of sample and a flow rate of 0.5 ml min^–1^. The mobile phase consisted of Solvent A, a 7:2:1 (water:methanol:acetonitrile) mixture, and Solvent B, a 3:1:1 (acetonitrile:MTBE:methanol) mixture. A gradient starting from 60% A and 40% B was followed by 25% A and 75% B for 5 min, and then 100% B for 15 min. After, the sample was held for 20 min. This was followed by a return to initial conditions (60% A, 40% B) held for a 6-min post-analysis equilibration. All spectra were captured at a range of absorbance from 300 to 950 nm, by a step of 4 nm, and a 0.62-Hz scan rate.

MS/MS analysis was performed using the Agilent 6540 UHD Q-Tof Mass Spectrometer, operating in positive ion mode. Mass spectra were obtained using the Agilent QTOF 6540 mass spectrometer equipped with the JetStream ESI ion source. The mass spectrometer was scanned from m/z 100 to 1,700 and operated in the 4-GHz HRes mode. Accurate mass measurement was achieved by constantly infusing a reference calibrant (ions at m/z 121.0508 and 922.0098). An Agilent Technologies 1100 Series HPLC system coupled to an Agilent Technologies Mass Selective Trap SL detector equipped with an atmospheric pressure chemical ionization source was used for MS^5^ analysis of selected Bchl molecules using directed infusion. All pigments were identified by comparison with published reference absorption spectra (Jensen, 1965; Airs and Keely, 2002).

### Microscopy

A 2-μl aliquot from the enrichment grown with Fe(II) was placed onto a carbon film grid (Electron Microscopy Sciences) and allowed to settle for 1 min. After additional liquid was wicked from the grid, 2 μl of aqueous 2% uranyl acetate was immediately added and allowed 30 s to fully immerse the grid. Following a final wick of the grid, it was allowed to dry leaving a thin film of cells. Transmission electron microscopy (TEM) images were obtained using JEOL 2100 STEM in the Roy J. Carver High Resolution Microscopy facility at ISU. The images were captured under normal high vacuum conditions at 200 kV with a Gatan OneView 4K camera. Fluorescence images were obtained as described in section “Growth Experiments With Fe(II).”

## Results and Discussion

### Enrichment of BLA1

Initial enrichment of lake water from the chemocline on FW medium containing Fe(II) with a long-pass light filter resulted in growth as assessed by visible Fe(II) oxidation and precipitation. After dilution-to-extinction and propagation for several generations, DNA was extracted, and the 16S rRNA gene was amplified and cloned to identify dominant taxa in the enrichment. All clones sequenced (17 of 17) were identical and had close matches to photoferrotrophic GSB.

**FIGURE 1:**
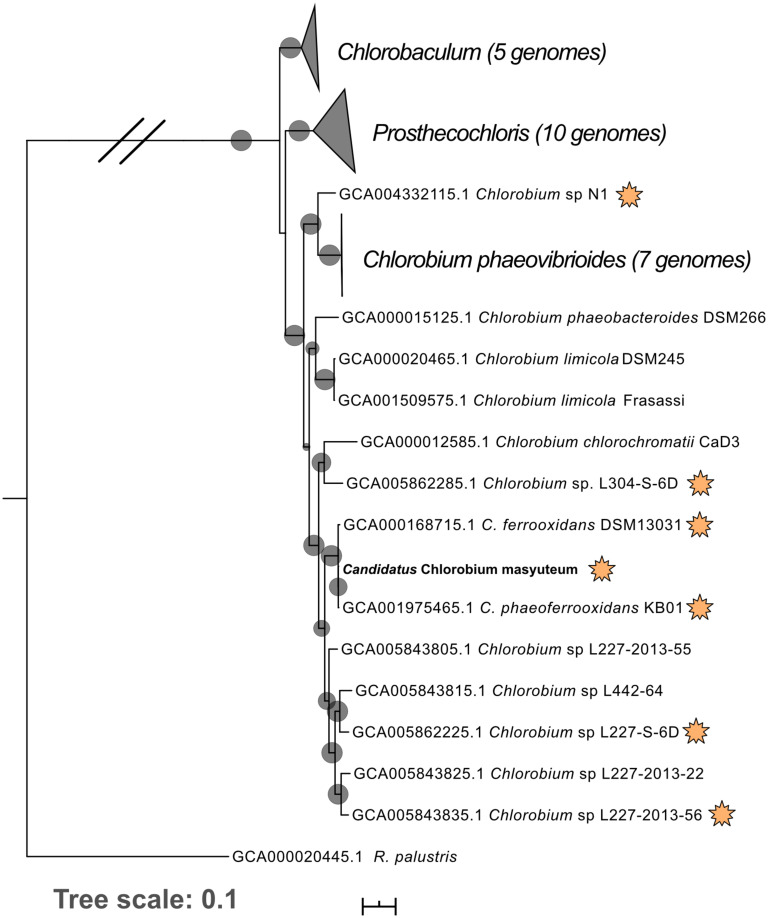
Phylogenomic tree of “*Ca*. C. masyuteum” with other Chlorobia isolates and metagenome assembled genomes. Genomes highlighted with orange stars are either experimentally verified or inferred from the genome to photo-oxidize Fe(II). Bootstrap values (>75%) are represented by black circles. Orange stars represent organisms with either tested or inferred photoferrotrophy capability.

**FIGURE 2:**
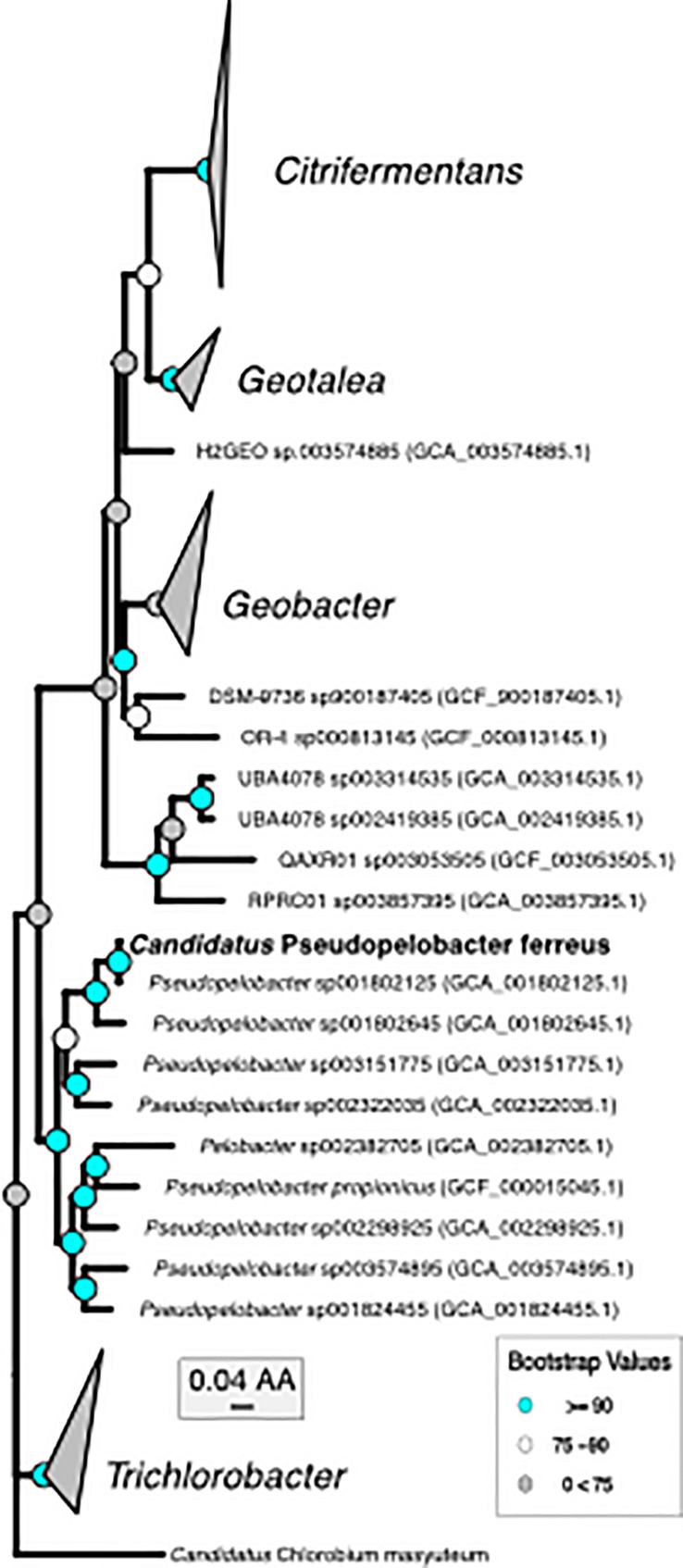
Phylogenomic tree of “*Ca.* P. ferreus” with the only other isolated organism in the genus and metagenome-assembled genomes of closely related organisms.

Metagenomics was then performed on the enrichment after dilution to extinction. The number of assembled contigs and total size of the assembly suggested more than one genome may be present. Searches of assembled 16S rRNA genes with Barrnap^2^ revealed two unique 16S rRNA sequences. The enrichment was subsequently named “BLA1,” as the first enrichment (A1) from Browne Lake (BL), and this epithet is applied to the dominant strain within the enrichment. The dominant genome (∼300 × coverage) was predicted to be 99% complete with 0% contamination. Taxonomic identification of the genome bin with GTDB-TK identified it as a novel species belonging to the *Chlorobium* genus based on average nucleotide differences. The 16S rRNA gene from the *Chlorobium* sp. BLA1 genome matched 100% by BLAST (Altschul et al., 1990) to the 16S rRNA clones which initially recovered “BLA1” enrichment. The recovered *Chlorobium* sp. BLA1 genome was closely related to other known photoferrotrophic GSB, particularly *C. ferrooxidans* and *C. phaeoferrooxidans* KB01 (Figure 1). The second and less abundant genome (4 × coverage) was 97% complete with 2% contamination. *Pseudopelobacter* sp. SKOL is taxonomically associated with the novel order Geobacterales and novel family *Pelobacteraceae* (Figure 2; Waite et al., 2020). GTDB-TK identified the genome as a novel species within the *Pseudopelobacter* genus and is similar to a preexisting genome, *Pseudopelobacter* sp001802125 (GTDB taxonomy, NCBI name GWC2_55_20).

### Morphology and Growth of BLA1

**FIGURE 3:**
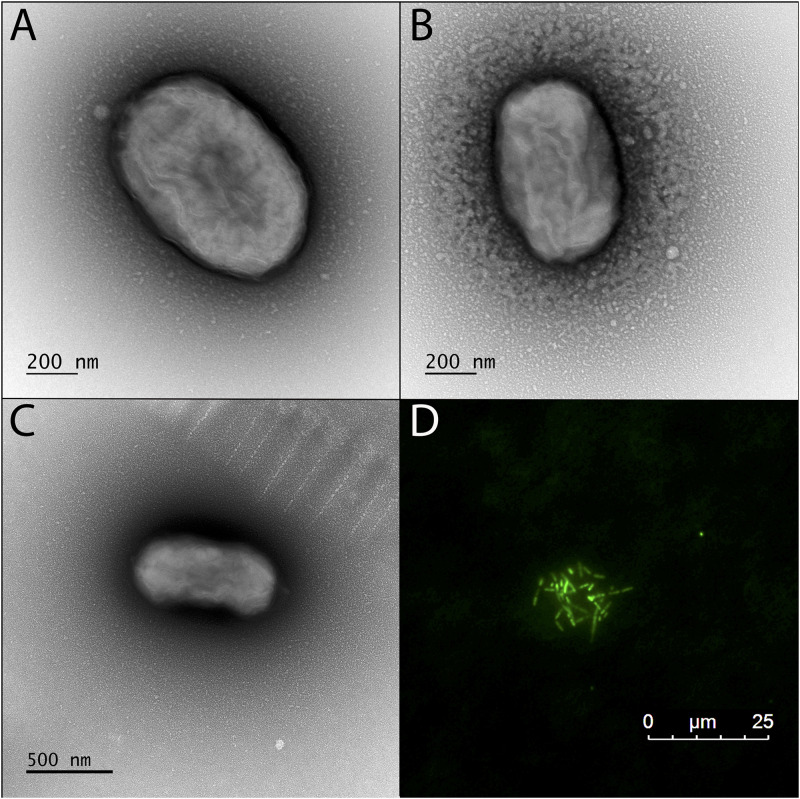
Transmission electron microscopy (TEM) and fluorescence microscope images of the BLA1 enrichment grown in FW medium with Fe(II). **(A–C)** TEM; cell surfaces appear wrinkled, while still maintaining the structure of the cell, due to dehydration during TEM preparation. **(D)** Fluorescence image of live cells clustered on an Fe(III) mineral.

TEM revealed that the dominant cell type in BLA1 was rod-shaped, ca. 0.8–0.9 μm long and 0.4–0.6 μm wide (Figure 3). Cells did not contain flagella and are immotile, consistent with all other photoferrotrophs assessed for motility (Ehrenreich and Widdel, 1994; Heising et al., 1999; Straub et al., 1999). Cell morphology appeared similar to *C. ferrooxidans* strain KoFox (Heising et al., 1999).

**FIGURE 4:**
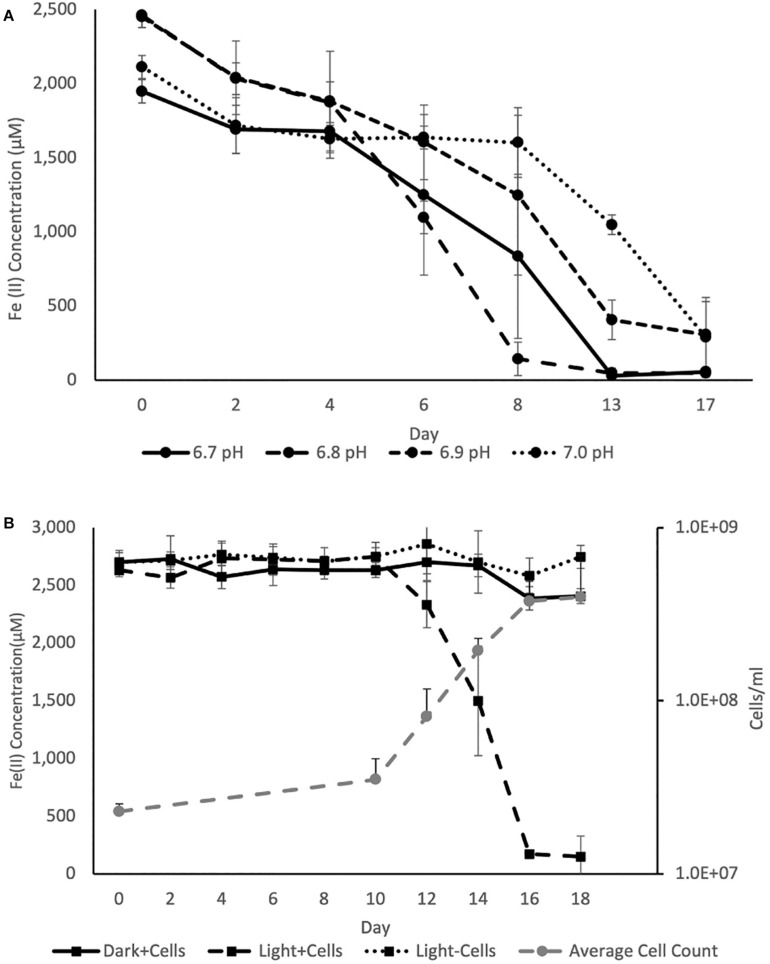
**(A)** Average Fe(II) oxidation curves for triplicate BLA1 incubations under illumination at different pH, showing optimal activity at pH 6.8. **(B)** Representative growth and Fe(II) oxidation of BLA1 from one triplicate. Error bars represent the standard deviation of analytical triplicates for Fe(II) (in all cases smaller than symbol size) and the standard deviation of all fields of view counted for cell counts.

The pH optimum for BLA1 growth on Fe(II) was pH 6.8 as determined by the Fe(II) oxidation rate (Figure 4). This is similar to the optimum for other photoferrotrophs (Straub et al., 1999), although a freshwater photoferrotrophic *Chlorobium* sp. isolated from Lake Constance had an optimum of pH 7.4–7.6 (Schmidt et al., 2021). Protons are a product of Eq. 1, which likely explains the preference of many photoferrotrophs for circumneutral pH. The pH of water decreases with depth through the chemocline of Brownie Lake and is seasonally variable, but is generally from 8 near the epilimnion down to 6.5 near the monimolimnion. Fe(II) oxidation rates were slowest at pH 7.0 (Figure 4), indicating that ideal conditions for this organism and Fe(II) oxidation may occur near the bottom of the chemocline.

The rate of Fe(II) oxidation in other photoferrotrophic organisms has also been shown to increase with higher Fe(II) concentrations and increasing light intensity (e.g., Michaelis–Menten kinetics; Hegler et al., 2008; Wu et al., 2014; Laufer et al., 2017). The effect of these additional factors on Fe(II) oxidation rate was not explored here. However, PAR in the chemocline of Brownie Lake varied from a maximum of 1–2 μmol photons m^–2^ s^–1^ at the top of the chemocline down to 0.1 μmol photons m^–2^ s^–1^. Such conditions are consistent with GSB being able to inhabit some of the lowest light environments of all photosynthetic bacteria (Overmann and Garcia-Pichel, 2013).

Iron oxidation was followed in triplicate incubations at pH 6.8 with an initial density of ∼2.2 × 10^7^ cells ml^–1^ and at a light intensity of 4.2 μmol photons m^–2^ s^–1^. Serum bottles of FW medium containing Fe(II) showed evidence of Fe(II) oxidation after 8–10 days. No oxidation of Fe(II) was observed in cultures incubated in the dark or in uninoculated samples incubated in the light (Figure 4). The average rate of Fe(II) oxidation at 20°C and 4.2 μM photons m^–2^ s^–1^ was 0.63 ± 0.069 mmol day^–1^. After 16 days, 95% of the Fe(II) was oxidized. The Fe(II) oxidation rate of BLA1 is commensurate with other photoferrotrophic enrichments containing *Chlorobium* sp., such as the marine *Chlorobium* sp. strain N1, 0.77 ± 0.02 mmol day^–1^ (Laufer et al., 2017). It is also similar to the freshwater purple non-sulfur *R. ferrooxidans* strain SW2, 0.40 mM day^–1^ (Kappler et al., 2005). Cell numbers increased under Fe(II) growth conditions to 3.8 × 10^8^ cells ml^–1^ at day 16 (Figure 4). The doubling time during Fe(II) oxidation was 0.6 days (14.3 h). This doubling time is comparable to *Chlorobium* sp. strain N1 (0.4 days or 9.6 h; Laufer et al., 2017) but is faster than *C. ferrooxidans* strain KoFox (5.3 days; Heising et al., 1999). At 3.8 × 10^8^ cells ml^–1^, the average cell-specific Fe(II) oxidation rate for *Chlorobium* sp. BLA1 was 1.68 ± 0.26 fmol cell^–1^ day^–1^. This is comparable to *Chlorobium* sp. strain N1 (1.15 fmol cell^–1^ day^–1^; Laufer et al., 2017), but ∼1,000 × lower than for photoferrotrophs inhabiting the chemocline of Kabuno Bay (1.25 pmol cell^–1^ day^–1^; Llirós et al., 2015).

Similar to many other enriched or isolated photoferrotrophic *Chlorobium* sp., the BLA1 enrichment did not show evidence for growth with reduced sulfur compounds (Table 1). The exception is *Chlorobium* sp. N1, which was enriched from marine sediments (Laufer et al., 2017) where sulfur compounds are generally more abundant than lake water. *Chlorobium* sp. from ferruginous lakes have sometimes also been determined to have the capacity for photosynthetic oxidation of both Fe(II) and reduced sulfur compounds (Crowe et al., 2014; Tsuji et al., 2020), perhaps indicating that selection for and utilization of photoferrotrophy in the environment is dependent on the specific environmental conditions, such as low sulfur. Brownie Lake, from which BLA1 was enriched, does have 50–100 μM sulfate and hydrogen sulfide is periodically detected in anoxic water (Lambrecht et al., 2018).

**TABLE 1:** Commonly used sole organic and inorganic electron donors to assess additional photoautotrophic growth of true photoferrotrophic isolates or enrichments.

Photoferrotroph	Iron(II)	Acetate	Hydrogen	Sulfide	Thiosulfate	Source
“*Ca.* C. masyuteum”	+	(+)	(+)	-	-	This study
*C. ferrooxidans* strain KoFox	+	-	+	-	-	Heising et al., 1999
						Hegler et al., 2008
*C. phaeoferrooxidans* strain KB01	+	n. d.	n. d.	n. d.	n. d.	Crowe et al., 2017
*Chlorobium* sp.	+	(+)	+	-	-	Schmidt et al., 2021
*Chlorobium* sp. strain N1	+	+	+	+	+	Laufer et al., 2017
*Thiodycton* sp. strains Thd2 and F4	+	+	+	-	-	Ehrenreich and Widdel, 1994
						Hegler et al., 2008
*R. palustris*strain TIE-1	+	+	+	-	+	Jiao et al., 2005
*R. ferrooxidans*strain SW2	+	+	+	-	n. d.	Ehrenreich and Widdel, 1994
*R. iodosum*	+	+	+	+	+	Straub et al., 1999
*R. rubiginosum*	+	+	+	+	+	Straub et al., 1999

**(+) indicates growth on this substrate may not be attributed solely to this strain. Thiodycton strain L7 in Ehrenreich and Widdel (1994) was given accession number X78718, and this accession number in GenBank recognizes this isolate as strain Thd2. n.d. = no data.**

The BLA1 enrichment was able to grow with both H_2_ and acetate (Table 1). This finding is very similar to a freshwater enrichment of a photoferrotrophic *Chlorobium* sp. with a photosynthetic *Rhodopseudomonas* sp. (Schmidt et al., 2021). In that study, detailed experiments and subsequent isolation of the *Rhodopseudomonas* sp. revealed that while only the *Chlorobium* sp. was capable of photosynthetic Fe(II) oxidation, both strains were capable of H_2_ oxidation, and only the *Rhodopseudomonas* sp. was capable of growth using acetate (Table 1). For BLA1, observable growth on H_2_ was seen approximately 10–14 days following inoculation, and growth on acetate often required a lag time of 8–14 days. Direct Sanger sequencing of the 16S rRNA gene amplified from DNA extracted when BLA1 was grown with H_2_ or acetate resulted in a clean sequence matching of the 16S rRNA recovered from the *Chlorobium* sp. BLA1 genome, suggesting robust growth by that organism on these substrates.

Nevertheless, there is the possibility for a close relationship with the *Pseudopelobacter* sp., much like what was observed by Schmidt et al. (2021) in their enrichment. It seems unlikely to involve simultaneous Fe(III) reduction by *Pseudopleobacter* sp. SKOL during Fe(II) oxidation in the absence of acetate (see below for a discussion of functional capabilities encoded in both genomes) as no Fe(III) reduction was observed after all Fe(II) had been oxidized (Supplementary Figure 1). However, acetate and formate are detectable in the chemocline of Brownie Lake, suggesting redox cycling of Fe between these two organisms could be possible. No H_2_ measurements have been made in Brownie Lake.

### Phylogenomics of BLA1 Enrichment Genomes

*Chlorobium* sp. BLA1 is > 99% similar by the 16S rRNA gene to *C. ferrooxidans* and *C. phaeoferrooxidans*, and the three genomes are similar in size, %GC, and gene number (Table 2). This result is congruent with the phylogeny of sequenced genomes (Figure 1). All three of these strains are genetically distinct from other anoxygenic phototrophic Chlorobia that lack the ability to oxidize Fe(II). For instance, the *Chlorobium* sp. BLA1 16S rRNA sequence is only 97% similar to that of *Chlorobium clathratiforme*. Therefore, to distinguish whether *Chlorobium* sp. BLA1 is a new species or a subspecies of either *C. ferrooxidans* or *C. phaeoferrooxidans*, we used two independent measures of genome relatedness, digital DNA–DNA hybridization (DDH) and average nucleotide identity (ANI). Both DDH and ANI values indicate that *Chlorobium* sp. BLA1 is a novel species (Table 2). DDH values for all the closest related genomes were well below the accepted cutoff of 70% (Meier-Kolthoff et al., 2013). Additionally, ANI values for these genomes were also below the 95% cutoff for species level (Jain et al., 2018). These results indicate that at the genome level, *Chlorobium* sp. BLA1 is sufficiently divergent from *C. ferrooxidans* and *C. phaeoferrooxidans* to be considered a distinct species. Thus, we assign the name “*Candidatus* Chlorobium masyuteum.”

**TABLE 2:** Isolate genome characteristics and comparisons to other *Chlorobia* genomes.

Reference genome	Strain name	DDH	Model C.I. (%)	ANI	%GC	Genome size (Mb)	No. genes
“*Ca.* Chlorobium masyuteum”	BLA1	–	–	–	50.6	2.56	2,312
*Chlorobium ferrooxidans*	DSM 13031	54.8	52.1–57.5	94.1	50.1	2.54	2,308
*Chlorobium phaeoferrooxidans*	KB01	51.5	48.9–54.2	93.4	49.7	2.57	2,486
*Chlorobium* sp.	L227-2013-55	19.9	17.7–22.3	78.9	48.5	2.38	2,338
*Chlorobium* sp.	L227-2013-22	19.9	17.7–22.3	78.7	45.9	2.55	2,459
*Chlorobium* sp.	L227-S-6D	18.9	16.8–21.3	78	46.8	2.49	2,383
*Chlorobium* sp.	L227-2013-56	18.5	16.3–20.9	77.6	43.9	2.89	1,991
*Chlorobium* sp.	L442-64	19.7	17.5–22.1	78.1	48.3	1.9	1,851
*Chlorobium phaeobacteroides*	DSM 266	19.2	17–21.6	77.6	48.4	3.13	2,819
*Chlorobium* sp.	N1	17.9	15.8–20.3	77.6	61.5	2.37	2,284

**Reference genome identifiers correspond to isolates in Figure 1.**

The second organism, *Pseudopelobacter* sp. SKOL, is taxonomically associated with the Order *Geobacterales* and was far less abundant in the assembly (4 × coverage). The genome is most closely related to *Pseudopelobacter sp001802645* (95.5% similar by ANI), a metagenome-assembled genome (MAG) from groundwater in Rifle, CO (Anantharaman et al., 2016). Currently, there is one isolated species, *Pseudopelobacter propionicus*, from this proposed genus. We propose the name “*Candidatus* Pseudopelobacter ferreus.”

### Predicted Functional Capability of BLA1 Genomes

#### “*Candidatus* Chlorobium masyuteum”

**FIGURE 5:**
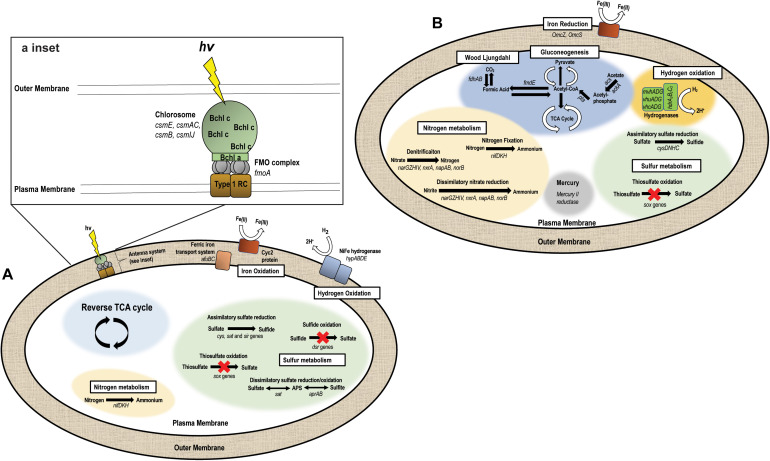
Metabolic pathways encoded by the two genomes recovered from the BLA1 enrichment. **(A)** “*Ca.* C. masyuteum” (APS—adenylyl sulfate). Inset shows the antenna system (RC—reaction center, FMO—Fenna–Matthews–Olson, Bchl—Bacteriochlorophyll). **(B)** “*Ca.* P. ferreus.”

The “*Ca.* C. masyuteum” genome consists of 17 contigs, with 2,312 predicted genes, and two rRNA operons. Gene and protein families predicted to function in key physiological activities of “*Ca.* C. masyuteum” are presented in Figure 5A. As with other members of the class Chlorobia, “*Ca.* C. masyuteum” has a type 1 photosynthetic reaction center (RC). Chlorosomes are unique membrane-bound photosynthetic antenna complexes found exclusively in GSB that contain large numbers of Bchl molecules (Imhoff, 2014). Chlorosome genes (*csmE*, *csmAC*, *csmB*, and *csmIJ*) appeared on four separate contigs. The *fmoA* gene encoding for the Fenna–Matthews–Olson protein are also present. The Fenna–Matthews–Olson protein participates in energy transfer from chlorosomes to the RC in the *Chlorobiaceae* (Fenna et al., 1974). *Chlorobiales* synthesize Bchl *a* and Chl *a*, as well as major Bchl (*c*, *d*, or *e*) to harvest light (Bryant and Liu, 2013). The genome of “*Ca.* C. masyuteum” contained at least six protein families corresponding to the synthesis of Bchl *a* and two corresponding to Chl *a* synthesis (Figure 5A and Supplementary Table 1). Additionally, two protein families specific to the Bchl *c* synthesis pathway, BchU and BchV, were present (Maresca et al., 2004; Chew, 2007).

The functional hallmark of the BLA1 enrichment is the ability to grow photoautotrophically using Fe(II). In photoferrotrophic purple bacteria, the *pioABC* operonis a three-gene operon coding for a periplasmic decaheme c-type cytochrome that transfers electrons (*pioA*), an outer membrane β-barrel protein (*pioB*), and a periplasmic high potential iron-sulfur cluster protein that participates in anaerobic electron transport (*pioC*; Jiao and Newman, 2007). This operon is found in *R. vannielii* strain ATCC 17100 (He et al., 2017) and *R. palustris* (Jiao and Newman, 2007). The *foxEYZ* operonis another three-gene operon that encodes for a c-type cytochrome (*foxE*), a putative protein containing the cofactor pyrroloquinoline (*foxY*), and a putative protein with transport function (*foxZ*) and is found in *R. ferrooxidans* (Croal et al., 2007). Annotation of the “*Ca.* C. masyuteum” genome indicates that the *pio* and *fox* operons are absent. This finding is similar to other GSB photoferrotrophs (Bryce et al., 2018; Tsuji et al., 2020), leading us to screen for other genes with potential Fe(II) oxidase activity.

Recently, homologs to an outer-membrane c-type cytochrome that functions in Fe(II) oxidation, encoded by the gene *cyc2*, have been detected in both acidophilic and neutrophilic oxygen-dependent Fe(II) oxidizing bacteria. *Acidothiobacillus ferrooxidans, Mariprofundus* sp., *Acidothiobacillus ferrooxidans, Mariprofundus* sp., *Gallionellaceae*, and *Zetaproteobacteria* are such examples (Castelle et al., 2008; Barco et al., 2015; Chan et al., 2018; McAllister et al., 2020). Subsequently, the *cyc2* gene was found in the genomes of *C. ferrooxidans* and *C. phaoferrooxidans* (He et al., 2017; Chan et al., 2018) and have been detected in *Chlorobium* metagenomes in enrichments from ferruginous lakes (Tsuji et al., 2020). We identified *cyc2* in the genome of “*Ca.* C. masyuteum.” In addition, we identified two genes, *afuBC*, which are integral and cytoplasmic membrane proteins of the ferric iron ABC transport system in “*Ca.* C. masyuteum” (Figure 5A).

The BLA1 enrichment was not able to oxidize sulfide, sulfite, or thiosulfate (Table 1). The “*Ca.* C. masyuteum” genome was in agreement with growth experiments, as genes implicated in sulfide (*dsr*) or thiosulfate (*sox*) oxidation were absent (Figure 5A). This differs from the GSB *Chlorobium* sp. strain N1 (Laufer et al., 2017) and the PSB *R. iodosum* and *R. rubiginosum* (Straub et al., 1999). *R. palustris* strain TIE-1 is able to oxidize thiosulfate, but no reports indicate the oxidation of sulfide by TIE-1 or closely related strains (Jiao et al., 2005; Schmidt et al., 2021). The ability to oxidize these sulfur compounds is also absent from the well-studied *C. ferrooxidans* strain KoFox (Heising et al., 1999). Genes implicated in sulfite oxidation, *aprAB* and *sat*, were present in the “*Ca.* C. masyuteum” genome but are likely also used for the assimilation of sulfur as no sulfite oxidation was observed. The *sat* genes are absent in other photoferrotrophic *Chlorobium* sp. (Thompson et al., 2017). No other sulfur oxidation genes were present.

In addition, the genome of “*Ca.* C. masyuteum” contains *cys*, *sat*, and *sir* genes which participate in assimilatory sulfate reduction (ASR). Other photoferrotrophic *Chlorobium* sp. (e.g., *C. phaeoferrooxidans*, *C. ferrooxidans*, “*Ca*. C. canadense” L304-6D, and L227 enrichment S-6D) have been shown to contain ASR genes, particularly *cys* (Thompson et al., 2017; Tsuji et al., 2020), and sulfate assimilation has been biochemically verified in the first two strains. During isolation of *Chlorobium* sp. strain N1, the authors suggested sulfate could be utilized as a sulfur source; however, ASR activity was not directly tested (Laufer et al., 2017).

Nitrogen fixation has been recognized in *Chlorobium* sp. for quite some time (Heda and Madigan, 1986; Bryant et al., 2012), and GSB may be important sources of fixed nitrogen in stratified water columns (Halm et al., 2009; Swanner et al., 2020). Although fixed nitrogen was always provided in the FW medium, the “*Ca.* C. masyuteum” genome does contain *nifDKH* genes encoding a Mo-requiring nitrogenase (Figure 5A). These genes have also been detected and growth without fixed nitrogen verified in the closely related photoferrotrophs *C. ferrooxidans* and *C. phaeoferrooxidans* (Thompson et al., 2017).

Genes necessary to fix carbon using the reverse tricarboxylic acid cycle typical for *Chlorobium* sp. are present in “*Ca.* C. masyuteum” (Tang and Blankenship, 2010). The BLA1 enrichment was also able to grow with acetate. As acetate utilization is an unusual capability for organisms within the *Chlorobiaceae* family (Imhoff, 2014), it is possible that this mode of growth may have required some type of syntrophy with “*Ca.* P. ferreus,” although acetate cultures were dominated by “*Ca.* C. masyuteum” based on Sanger sequencing. Other photoferrotrophic *Chlorobium* that are able to use acetate but only in coculture are *Chlorobium ferrooxidans* (strain KoFox) with *Geospirillum sp.* strain Kofum (Heising et al., 1999) and the closely related *Chlorobium* sp. obligately in coculture with acetate utilizing *Rhodopseudomonas* sp. (Schmidt et al., 2021). Considering we did observe robust growth dominated by “*Ca.* C. masyuteum” on acetate, we suggest it could also be possible for “*Ca.* C. masyuteum” to use its reverse TCA cycle or run the reverse TCA in the forward direction to assimilate acetate, such as has been observed for *C. tepidum* (Tang and Blankenship, 2010).

Oxidation of H_2_ for photoautotrophic growth requires membrane-bound NiFe hydrogenases (Schwartz et al., 2006). The “*Ca.* C. masyuteum” genome contains *hypABDE* genes encoding for a Group 1d Ni-Fe hydrogenase (Figure 5A), similar to the genomes of *C. ferrooxidans* and *C. phaoferrooxidans*. This, along with growth experiments, suggests that “*Ca.* C. masyuteum” is able to grow photoautotrophically utilizing H_2_. However, growth on H_2_ was not observed by *C. phaoferrooxidans* despite the presence of *hyp* genes (Thompson, 2020). Further work to establish whether *hypABDE* genes encode photosynthetic hydrogen oxidation may help to determine which genes can be used as genetic markers for photosynthetic hydrogen oxidation by GSB in environmental contexts (Thompson, 2020; Tsuji et al., 2020; Garcia et al., 2021).

#### “*Candidatus* Pseudopelobacter ferreus”

The “*Ca.* P. ferreus” genome is 4.5 Mb, consists of 139 contigs, with 4,019 predicted genes, and one 16S rRNA gene, and has a GC content of 54.6%. Although it is not photosynthetic, this organism is metabolically flexible, with gene and protein families predicted to function in transformation of C, Fe, N, S, H, and Hg (Figure 5B). The “*Ca.* P. ferreus” genome encodes for the reductive acetyl-CoA or Wood–Ljungdahl pathway for carbon fixation common among the Delta Proteobacteria (Hügler and Sievert, 2011).

“*Ca.* P. ferreus” seems likely to function as an FeRB when grown in the BLA1 enrichment, considering that the genome contains gene homologs for dissimilatory Fe(III) reduction and the detection of Fe(III) reduction activity in BLA1 after complete Fe(II) oxidation and acetate addition (Supplementary Figure 1). These homologs include *omcS* and *omcZ*, putatively thought to be involved in long-distance extracellular electron transfer (Santos et al., 2015; Wang et al., 2019), and additional hypothetical proteins involved in electron transfer including porins, periplasmic cytochromes, and outer-membrane cytochromes (Garber et al., 2020). The low abundance of the “*Ca.* P. ferreus” genome in the BLA1 enrichment in combination with no subsequent Fe(III) reduction before acetate was added suggests that Fe(III) reduction is likely not occurring during photoferrotrophic growth in the absence of added electron donors.

Fe(III) reduction by “*Ca.* P. ferreus” can likely be coupled to oxidation of H_2_. The genome encodes for the cytoplasmic heterodisulfide reductase (HdrABC) as well as a NiFe hydrogenase (MvhAGD). This complex seems to function in H_2_ oxidation and possibly electron bifurcation in a number of anaerobes, including methanogenic Archaea and *Geobacter sulfurreducens* (Wischgoll et al., 2005; Wagner et al., 2017). Fe(III) reduction activity with H_2_ was not assessed for BLA1.

Although it is capable of autotrophy, “*Ca.* P. ferreus” encodes multiple pathways for the incorporation of organics. Since BLA1 grew when amended with acetate, we have focused on possible pathways to explain this phenomenon (Figure 5B). Acetate could be incorporated as acetyl phosphate (*via acs* and *ackA*) and converted to acetyl-CoA (*pta*). Acetyl-CoA could then feed into the TCA cycle or gluconeogenesis after conversion to pyruvate. Another possibility is that acetate is incorporated as acetyl-CoA by reversing the Wood–Ljungdahl pathway, such as occurs in some methanogens and anaerobes (Zhuang et al., 2014).

In addition to its potential role in Fe(III) reduction, the genome of “*Ca.* P. ferreus” also encodes a mercury(II) reductase. Similar functionality has been detected in other Geobacterales (Lu et al., 2016). The genome also encodes for Mo-requiring nitrogenase *via nifDKH* and contained genes for ASR (i.e., *cysDNHC*).

### Pigments Produced by “*Ca.* C. masyuteum”

**FIGURE 6:**
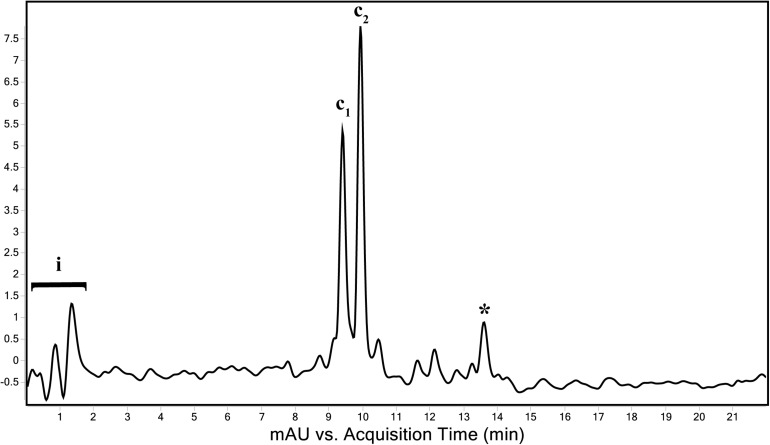
Total wavelength chromatogram depicting the major pigments of “*Ca.* C. masyuteum.” i—sample injection peaks; c_1_ and c_2_—bacteriopheophytins corresponding to bacteriochlorophyll *c*; *chlorobactene.

**FIGURE 7:**
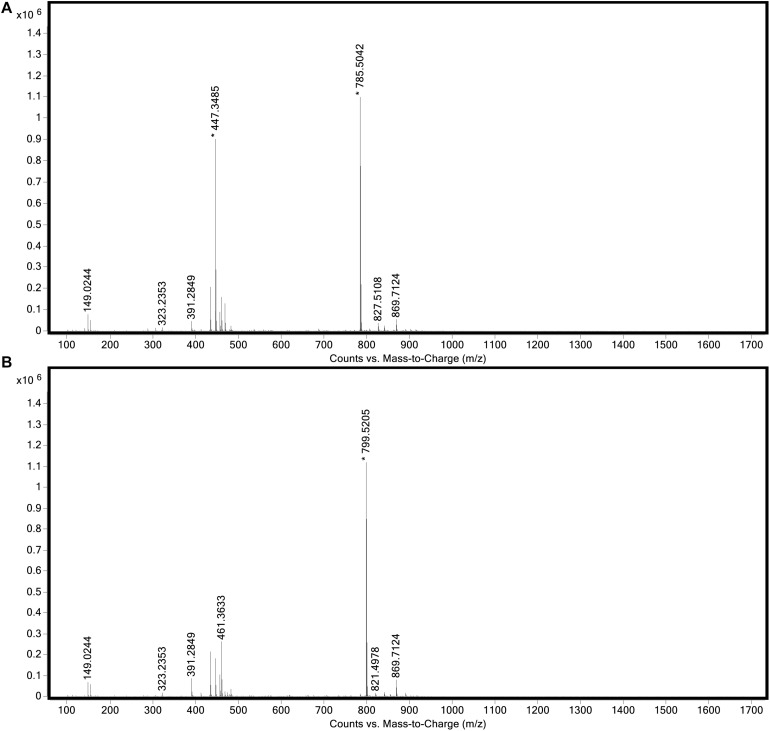
Full mass spectra of **(A)** peak c_1_ and **(B)** peak c_2_ obtained during LC/MS analysis of the extract from “*Ca.* C. masyuteum.”

The genome of “*Ca.* C. masyuteum” consisted of protein families for important minor pigments Chl *a* and Bchl *a*, as well as the major light-harvesting pigment Bchl *c*. To confirm that Bchl *c*, as opposed to Bchl *d* or *e*, is the major light-harvesting pigment, an extract from the BLA1 enrichment grown on Fe(II) was examined using a Q-TOF LC/MS system. The resulting chromatogram (Figure 6) displayed three major peaks corresponding to light-harvesting molecules. The first peak, denoted as c_1_, eluted between 9.3 and 9.7 min and gave rise to a major ion at *m/z* 785 (Figure 7A). The second peak, denoted as c_2_, eluted later between 9.9 and 10.2 min and displayed an increasing mass difference of 14 Da (*m/z* 799; Figure 7B). Peaks of low relative abundance were detected in the mass spectra indicating the presence of numerous background ions. As “*Ca.* C. masyuteum” was the dominant organism under these growth conditions, and *“Ca.* P. ferreus” is non-photosynthetic, we interpret the detected pigments as sourcing from “*Ca.* C. masyuteum.”

Peaks c_1_ and c_2_ were identified as bacteriopheophytins (Bphes), suggestive of two structural isomers of Bchl *c* based on previous identification with similar mass spectra (Airs and Keely, 2002). The detection of Bphes in place of Bchl *c* is likely due to post-column demetallation of the center Mg^2+^ (Table 2; Airs and Keely, 2002). The verification of the Bphes peaks as derivatives of Bchl *c* was assessed by performing a MS^5^ analysis, which creates fragmentation patterns unique to Bchl *c*, *d*, or *e*. The fragmentation pattern was as compared to work performed by Airs and Keely (2002) and indicative of Bchl *c* (Supplementary Figure 2A). The corresponding UV/vis spectrum agreed with the MS^5^ analysis, which depicted major absorption peaks characteristic of Bchl *c* at 412 and 668 nm (Supplementary Figure 2B). This is similar to what has been observed by Airs and Keely (2002). However, the peak at 412 nm is shifted to the left (412 nm instead of 435 nm) of what was observed by Airs and Keely (2002), likely due to the loss of the Mg^2+^ ion. The utilization of Bchl *c* as the main light-harvesting pigment is yet another feature that distinguishes “*Ca.* C. masyuteum” and its close relatives. *C. ferroooxidans* synthesizes Bchl *c* as well (Heising et al., 1999). However, *C. phaoferrooxidans* synthesizes Bchl *e* (Thompson, 2020).

The last of the major peaks eluted between 13.6 and 13.8 min (Figure 7). LC/MS analysis detected two molecules with overlapping retention times at this peak. Both molecules, γ-carotene and chlorobactene, likely contributed to the absorbance peak at ∼13.7 min. However, MS/MS fragmentation revealed major ions for chlorobactene [*m/z* 532.4 (radical) and *m/z* 533*z* 533.4 (M+H^+^)] and minor ions for γ-carotene [*m/z* 536.4 (radical) and *m/z 537.4* (M+H^+^); Supplementary Figure 3A]. The UV/vis spectrum of the major MS/MS fragmentation peak had three absorption maxima at 437, 460, and 488 nm (Supplementary Figure 3B). This sequence, when compared to reference absorption spectra (Jensen, 1965), corresponded to the aromatic carotenoid chlorobactene. The presence of both Bchl *c* and the accessory pigment chlorobactene in the extract agrees with previous work on GSB (Maresca et al., 2008). A similar absorption spectrum for chlorobactene was observed in extracts from the photoferrotroph *C. ferrooxidans* strain KoFox (Hegler et al., 2008).

The activity of photoferrotrophs in ferruginous oceans prior to the GOE has been implicated in the formation of massive Fe-Si deposits known as banded iron formations (BIF; Konhauser et al., 2002; Kappler et al., 2005; Hegler et al., 2008; Bekker et al., 2010; Mloszewska et al., 2012; Posth et al., 2013). However, ambiguity in assigning specific stable isotopic signatures exclusively to photoferrotrophy (e.g., Fe, see Swanner et al., 2015; Wu et al., 2017) indicates a need for other indicators of these organisms and their activity. Biomarkers are organic molecules that resist degradation and alteration and can be indicative of certain taxonomic groups (e.g., Bchl, aromatic carotenoids). Recent work has suggested that certain isoprenoids can act as biomarkers, linking APB to phototrophic Fe(II) oxidation in a Mesoproterozoic iron formation (Xiamaling Formation; 1.4 Ga) that shares similarities to Archean-aged BIFs (Canfield et al., 2018).

Chlorobactene exists as a monoaromatic carotenoid associated with green-pigmented GSB (i.e., *Chlorobiaceae*) and has been utilized as a proxy for GSB in the rock record as far back as 1.64 Ga (e.g., Barney Creek Formation; Summons and Powell, 1986). The presence of chlorobactene in the Barney Creek Formation, as well as other Precambrian deposits mentioned previously, has typically signified euxinic conditions. However, GSB phototrophs such as “*Ca.* C. masyutem” are capable of oxidizing iron but not reduced sulfur compounds (Table 1), and thus chlorobactene (or its derivatives) may not be a definitive indicator of euxinia. However, if found in combination with paleoredox proxies that indicate ferruginous conditions, detection of chlorobactene might serve as a line of evidence in support of photoferrotrophy. Continued effort is needed to better describe the environmental conditions under which sulfur vs. Fe(II)-oxidizing phototrophs are active, as cryptic phototrophic sulfur cycling has been detected in ferruginous environments (Crowe et al., 2014), and cryptic phototrophic iron cycling is noted from euxinic environments (Berg et al., 2016).

## Conclusion

In summary, two novel organisms were co-enriched from ferruginous meromictic Brownie Lake, Minnesota, United States. The most abundant organism in the BLA1 enrichment is “*Ca.* C. masyuteum,” a photoferrotrophic GSB that is genetically and physiologically unique when compared to close relatives. It is able to grow photoautotrophically using Fe(II), and possibly acetate and H_2_. It produces bacteriochlorophyll c as well as chlorobactene. The presence of the *cyc2* gene in “*Ca.* C. masyuteum” adds compounding evidence that this gene can be used as a marker for Fe(II) oxidation in GSB in ferruginous environments. The lack of sulfur oxidation pathways in this organism or observed reduced sulfur oxidation in the enrichment suggests that it likely performs photoferrotrophy in the environment but may also be able to use fermentation by-products like H_2_ or organic acids as electron donors for photosynthesis. The genome also encodes for a Mo-utilizing nitrogenase, suggesting a role for alleviation of nitrogen fixation in the redoxcline of Brownie Lake (Swanner et al., 2020).

“*Ca.* C. masyuteum” could not be isolated, and metagenomic sequencing revealed that a novel and metabolically flexible organism comprised about 1% of the BLA1 enrichment. “*Ca.* P. ferreus” is a putative FeRB that likely oxidizes acetate and/or H_2_. Two other photoferrotrophic GSB also live in defined coculture (Heising et al., 1999; Bryce et al., 2019), suggesting that close associations with other organisms may be a common strategy among photoferrotrophic GSB.

**Description of** “*Candidatus* Chlorobium masyuteum” (ma.syu.te for the phrase *mas’yúte*, meaning “eats iron” in the Dakota language spoken by the first caretakers of Brownie Lake). Short rod-like bacterium (0.8 μm by 0.4–0.6 μm in size). Selective enrichment from freshwater at 20°C with a long-pass light filter (i.e., > 700 nm). Grows autotrophically in freshwater medium with Fe(II) or molecular hydrogen as electron donors, in defined coculture with a *Pseudopelobacter* sp. Basis of assignment: digital DDH and ANI relatedness measures indicate a significant divergence at the genome to level from its closest *Chlorobium* relatives. Belongs to class Chlorobia, order *Chlorobiales*, and family *Chlorobiaceae*. Identified from a water sample of Brownie Lake, Minneapolis, Minnesota, United States.

**Description of “***Candidatus* Pseudopelobacter ferreus” (fer’re.us. L. masc. adj. ferreus pertaining to iron). Selective enrichment from freshwater at 20°C. Grows in anoxic freshwater medium in defined coculture with “*Ca.* C. masyuteum.” Basis of assignment: digital DDH and ANI relatedness measures indicate a significant divergence at the genome to level from its closest *Pseudopelobacter* relatives. Belongs to proposed phylum Desulfurbacterota, Desulfuromonadia class nov., *Geobacterales* order nov., and *Pseudopelobacteraceae* fam. nov. (Waite et al., 2020). Identified from a water sample of Brownie Lake, Minneapolis, Minnesota, United States.

## Data Availability Statement

The datasets presented in this study can be found in online repositories. The names of the repository/repositories and accession number(s) can be found in the article/Supplementary Material.

## Author Contributions

NL, ZS, and ES wrote the manuscript with input from all authors. NL and ES collected the field samples. MP, NL, ZS, and HT conducted the laboratory experiments with the isolate. CS, ZS, and NL analyzed and annotated the genomes, and created the figures and tables for the manuscript.

## Conflict of Interest

The authors declare that the research was conducted in the absence of any commercial or financial relationships that could be construed as a potential conflict of interest.

## Footnotes

1
http://hmmer.org/

2
https://github.com/tseemann/barrnap
