# Corrigendum: “*Candidatus* Chlorobium masyuteum,” a Novel Photoferrotrophic Green Sulfur Bacterium Enriched From a Ferruginous Meromictic Lake

**DOI:** 10.3389/fmicb.2021.758328

**Published:** 2021-09-07

**Authors:** Nicholas Lambrecht, Zackry Stevenson, Cody S. Sheik, Matthew A. Pronschinske, Hui Tong, Elizabeth D. Swanner

**Affiliations:** ^1^Department of Geological and Atmospheric Sciences, Iowa State University, Ames, IA, United States; ^2^Department of Biology, University of Minnesota Duluth, Duluth, MN, United States; ^3^Large Lakes Observatory, University of Minnesota Duluth, Duluth, MN, United States; ^4^Guangdong Key Laboratory of Integrated Agro-environmental Pollution Control and Management, National-Regional Joint Engineering Research Center for Soil Pollution Control and Remediation in South China, Guangdong Institute of Eco-environmental Science and Technology, Guangdong Academy of Sciences, Guangzhou, China

**Keywords:** photoferrotrophy, Brownie Lake, meromictic, green sulfur bacterium, phototrophic Fe(II) oxidation, early Earth biogeochemistry, iron cycling, geomicrobiology

In the original article, there was an error. **Some contributions were incorrectly acknowledged**.

A correction has been made to ***Acknowledgements*:**

Chad Wittkop and Sergei Katsev helped to collect water samples. We acknowledge the W. M. Keck Metabolomics Research Laboratory (Office of Biotechnology, Iowa State University) for providing analytical instrumentation and we thank Lucas Showman for his assistance and support. Tracey Stewart at the Roy J. Carver High Resolution Microscopy Facility at Iowa State University facilitated TEM sample preparation and imaging. Šišóka Dúta and Alexander Hall assisted with developing the species epithets. The Minneapolis Parks and Recreation Board supplied permits to work at Brownie Lake. This manuscript was originally part of a dissertation accepted by Iowa State University and edited for publication.

The authors apologize for this error and state that this does not change the scientific conclusions of the article in any way. The original article has been updated.

## Publisher's Note

All claims expressed in this article are solely those of the authors and do not necessarily represent those of their affiliated organizations, or those of the publisher, the editors and the reviewers. Any product that may be evaluated in this article, or claim that may be made by its manufacturer, is not guaranteed or endorsed by the publisher.

