# Supplementary material for: “Candidatus Chlorobium masyuteum,” a Novel Photoferrotrophic Green Sulfur Bacterium Enriched From a Ferruginous Meromictic Lake
Source: Front Microbiol. 2021 Jul 9;12:695260. doi: 10.3389/fmicb.2021.695260 (PMC8302410; doi:10.3389/fmicb.2021.695260)
Supplement: Supplementary file 1 [file Data_Sheet_1.docx]

*Supplementary Material*

*‘Candidatus* Chlorobium masyteum’, a novel photoferrotrophic green sulfur bacterium enriched from a ferruginous meromictic lake

Nicholas Lambrecht^1^, Zackry Stevenson^1^, Cody S. Sheik^2,3^, Matthew A. Pronschinske^1^, Hui Tong^1,4^, Elizabeth D. Swanner^1*^

^1^Iowa State University, Department of Geological and Atmospheric Sciences, Ames, IA, USA

^2^Department of Biology, University of Minnesota Duluth, Duluth, MN, USA

^3^Large Lakes Observatory, University of Minnesota Duluth, Duluth, MN, USA

^4^National-Regional Joint Engineering Research Center for Soil Pollution Control and Remediation in South China, Guangdong Key Laboratory of Integrated Agro-environmental Pollution Control and Management, Guangdong Institute of Eco-environmental Science & Technology, Guangdong Academy of Sciences, Guangzhou, China

# Supplementary Tables

**Supplementary Table 1**. Protein families present in the genome of ‘*Ca.* Chlorobium masyuteum’ as identified by metaErg that putatively function in harvesting light energy.

| **Pathway** | **Protein families** |
| --- | --- |
| Light-independent (dark-active) enzyme (DPOR)^†^ | ChlB  ChlL  ChlN |
| Major antenna pigments (Bchl *c, d, e*) ^‡^ | BchR  BchQ |
| Bchl *a* synthesis | BchC  BchF  BchG  BchP  BchX  BchY |
| Chl *a* synthesis | ChlG  ChlP |
| Bchl *c* synthesis | BchU  BchV |

† - Enzyme inclusive of all anoxygenic bacteria

‡ - Protein families involved in the biosynthesis of the included pigments


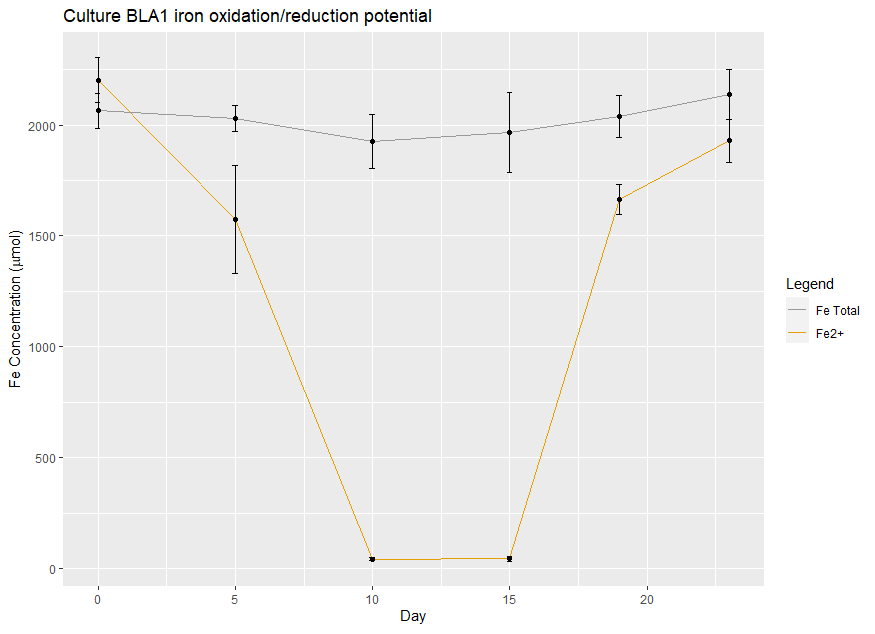


**Supplementary Figure 1.** Fe(II) oxidation and Fe(III) reduction during cultivation of BLA1. Day 0-15: Fe(II) is fully oxidized during light incubation. Day 15: Bottles are moved to the dark and 5 mM acetate was added. Fe(III) was subsequently reduced to Fe(II). Data is averaged from triplicates experiments, with error bars representing the standard deviation of all bottles.

**Supplementary Figure 2.**MS^5^ analysis (*m/z* 799>595>577>549) including the (a) fragmentation pattern and (b) a representative UV/VIS spectrum of the bacteriochlorophyll *c* molecule.

**Supplementary Figure 3.**MS/MS analysis of the peak eluting at ~13.7 minutes including the (a) fragmentation pattern and (b) the UV/VIS spectrum of the major MS/MS fragmentation peak.
